# Epigenetic Research of Neurodegenerative Disorders Using Patient iPSC-Based Models

**DOI:** 10.1155/2016/9464591

**Published:** 2015-11-30

**Authors:** Rubén Fernández-Santiago, Mario Ezquerra

**Affiliations:** ^1^Laboratory of Neurodegenerative Disorders, Department of Clinical and Experimental Neurology, Hospital Clinic of Barcelona, Institute of Biomedical Research August Pi i Sunyer (IDIBAPS), University of Barcelona (UB), 08036 Barcelona, Spain; ^2^Centre for Networked Biomedical Research in Neurodegenerative Disorders (CIBERNED), 28031 Madrid, Spain; ^3^Cell Therapy Program, Faculty of Medicine, University of Barcelona (UB), 08036 Barcelona, Spain

## Abstract

Epigenetic mechanisms play a role in human disease but their involvement in pathologies from the central nervous system has been hampered by the complexity of the brain together with its unique cellular architecture and diversity. Until recently, disease targeted neural types were only available as postmortem materials after many years of disease evolution. Current in vitro systems of induced pluripotent stem cells (iPSCs) generated by cell reprogramming of somatic cells from patients have provided valuable disease models recapitulating key pathological molecular events. Yet whether cell reprogramming on itself implies a truly epigenetic reprogramming, the epigenetic mechanisms governing this process are only partially understood. Moreover, elucidating epigenetic regulation using patient-specific iPSC-derived neural models is expected to have a great impact to unravel the pathophysiology of neurodegenerative diseases and to hopefully expand future therapeutic possibilities. Here we will critically review current knowledge of epigenetic involvement in neurodegenerative disorders focusing on the potential of iPSCs as a promising tool for epigenetic research of these diseases.

## 1. Introduction

A major challenge to model neurodegenerative disorders has been the inaccessible nature of the specific neural cell types targeted by disease which are usually available only in postmortem state. Recent somatic cell reprogramming protocols have contributed to overcome such a difficulty. Reprogramming of somatic cells to pluripotency can be currently achieved by different methods including somatic cell nuclear transfer (SCNT), fusion of somatic and pluripotent cells, included ectopic expression of defined sets of pluripotency transcription factors (TF) in adult somatic cells to generate induced pluripotent stem cells (iPSCs), and direct reprogramming of adult somatic cells to induced neurons (iN) by empirically determined cocktails of neurogenic factors [1–5]. In neurodegenerative disorders where animal models have not been able to entirely recapitulate key disease pathological aspects [6], reprogramming of human fibroblasts into iPSC has become a widely used technique permitting the generation of patient-specific disease-relevant cells in virtually limitless amounts with implications for the elucidation of disease mechanisms [7].

Parkinson disease (PD) is a neurodegenerative disorder associated with the progressive loss of dopaminergic neurons (DAn) in the substantia nigra pars compacta (SNpc) resulting in the cardinal motor symptoms of bradykinesia, rigidity, tremor, and postural instability [8, 9]. Due to their potential applicability for cell-based therapies, midbrain DAn were one of the first cell types generated by somatic cell reprogramming [10]. The resemblance of iPSC-derived DAn with midbrain DAn has centered various studies [11–13]. The hallmark of amyotrophic lateral sclerosis (ALS) is the selective death of motoneurons (MN) in the motor cortex, brain stem, and spinal cord leading to the progressive wasting and weakness of limb, bulbar, and respiratory muscles [14, 15]. Similar to DAn in PD, the high specialization and relative reduced number of MN in ALS also hold great potential for the use of somatic cell reprogramming in ALS cell-based therapies. Huntington disease (HD) is a monogenic neurodegenerative disorder triggered by trinucleotide expansions in the huntingtin gene causing corticostriatal dysfunction and leading to abnormal muscle coordination (choreic movements), mental decline, and behavioral symptoms [16, 17]. Alzheimer disease (AD) is a progressive neurodegenerative disorder characterized by global cognitive decline involving memory, orientation, judgment, and reasoning deficits which are associated with the deposition of amyloid plaques and neurofibrillary tangles in different brain areas including the hippocampus [18]. For these diseases and similar age-related neurodegenerative disorders, cell reprogramming has appeared as a promising tool to investigate the molecular and cellular processes related to the pathophysiological process in a subject-personalized manner.

Whereas only 5–10% of cases with neurodegenerative disorders such as AD, PD, or ALS are Mendelian disorders caused by pathogenic mutations in disease-associated genes, the vast majority of cases are considered sporadic resulting from the complex interplay of genetic risk factors and largely unknown environmental conditions [19, 20]. However, cumulative evidence has demonstrated that both monogenic and sporadic cases can share common pathogenic mechanisms [19]. To date, iPSC-derived neural models of PD [21–30], ALS [31–37], HD [38–41], and AD [42–45] have proved instrumental to model in vitro molecular alterations involved relevant to disease. Yet whether reprogramming of adult somatic cells constitutes a truly epigenetic reprogramming [7], detailed epigenomic characterization of patient-specific iPSC-based disease models and the role of epigenetic changes in the pathophysiology of these diseases remain underexplored. While it is well established that epigenetic alterations contribute to the physiopathology of human disease [46] including also neurodegenerative disorders [47], it should be accordingly expectable that iPSC-derived neural models may represent valid tools to investigate epigenetic changes involved in neurodegenerative disorders.

Here we will review the potentiality and current challenges of iPSC-based models to investigate epigenetic regulation of neurodegenerative disorders in the context of other existing patient specimens and disease models.

## 2. Epigenetic Mechanisms Relevant to the Nervous System

There are three major categories of epigenetic modifications including DNA methylation, histone posttranslational modifications, and noncoding RNAs (ncRNAs) which encompass microRNAs (miRNA), small noncoding RNAs (sncRNA), and long noncoding RNAs (lncRNA) [48]. The first two categories involving chemical changes in the DNA or histones will be discussed in this review. Regarding posttranslational modifications, which can mainly occur via acetylation, methylation, phosphorylation, and ubiquitination of histone residues, we will focus on histone acetylation and methylation since these two marks are relatively better characterized.

### 2.1. Epigenetic Definitions

“Epigenetics” can be defined as mitotically and/or meiotically heritable changes in gene expression which occur without changes in the DNA sequence. These epigenetic modifications mediate the execution of cell-type specific genomic programs activated in response to interoceptive as well as environmental stimuli. Conceptually, epigenetic mechanisms include long-term silencing, transcription, posttranscriptional RNA processing, translation, X chromosome inactivation, genomic imprinting, DNA replication and repair, and the maintenance of genomic integrity [49]. These epigenetic mechanisms play a crucial role in the regulation of gene expression by organizing the disposition of chromosomes in the nucleus, restricting or facilitating the access of TFs to DNA, and preserving a memory of past transcriptional activities [50]. A more operational definition of epigenetics is the study of heritable changes in gene activity caused by direct modification of the DNA sequence without altering the nucleotide sequence, namely, DNA methylation and posttranslational histone modifications [51]. For extension, the “epigenome” can be defined as the overall genomic collection of the DNA methylation states and covalent modification of histone proteins along the genome which is characteristic of each cell type [52]. Similarly, the specific epigenetic signature of each cell type is often designated as “epigenetic landscape.” The epigenetic signature of a particular cell, albeit normally stable, can also be dynamic over time [53] and thus dynamic changes in the epigenetic signature are known as “epigenetic plasticity.” According to the epigenetic theory, the genome and the environment can work synergistically impacting the regulatory mechanisms controlling gene expression by modification of the DNA epigenetic marks [54] which can occur throughout lifespan [55]. In monozygotic twins, epigenetic differences from the original epigenome have been shown to accumulate over time [56] and such divergences are known as “epigenetic drift.” This apparently stochastic accumulation of epigenetic changes has been associated with aging [57] and also with sporadic neurodegenerative disorders [58] in which, to date, aging is the major known risk factor [20].

### 2.2. DNA Methylation and Hydroxymethylation

DNA methylation is an important regulatory mechanism of gene expression in eukaryotes. In mammals and humans DNA methylation mostly affects the cytosine (C) base when it is followed by a guanine (G). These CpG sites can be clustered in the so-called “CpG islands” (GCI) but DNA methylation can also be present in non-CG contexts (mCH where H = A, C, or T). Both CpG and non-CG methylation are detected in the mouse and human brain [59, 60] but non-CG methylation is rare or absent in other differentiated cell types [61, 62]. Normally, DNA methylation follows a bimodal distribution ranging from unmethylated to fully methylated loci but intermediately methylated transition loci also exist. The methylation status of specific CpGs can be variable across individuals but stable over time within the same individual [63]. The biochemical process of DNA methylation involves the covalent modification of cytosines by addition of methyl groups (–CH_3_) to the 5′ position resulting in 5-methylcytosine (5mC). This reaction is catalyzed by specific enzymes termed de novo DNA methyltransferase (DNMT) and it occurs at expense of ATP and S-adenosylmethionine as methyl donor.

DNMT is expressed during neural development and in adult brain in a tissue- and cell-specific manner including areas of active neurogenesis [64] and adult stem cell niches [65] where they have been involved in neural survival and plasticity [66]. DNMT1 is the maintenance enzyme of DNA methylation sustaining methylation after DNA replication [67] whereas DNMT3a and DNMT3b have the capacity for methylating DNA de novo [68]. Of these, DNMT3b has been specifically involved in the specification of the neural crest [69]. Once methylation is established, proteins of the methyl-CpG-binding domain (MBD) family are recruited to methylated loci to elicit the recruitment of histone modulatory factors such as histone deacetylases (HDAC) [70, 71] indicating a synergistic coordination of different epigenetic marks [48]. The MBD proteins have also been involved in developmental and adult brain functions [72]. The most common consequence of DNA methylation is the silencing of genes and noncoding genomic regions, especially when affecting gene promoters. But DNA methylation can also be associated with enhanced expression by mechanisms that yet remain poorly understood [72, 73]. Recent studies have shown that about 75% of the DNA methylation affecting the gene body is associated with gene expression downregulation whereas the remaining 25% is associated with upregulation [74].

Other enzymes such as the oxidoreductases of the Ten-Eleven Translocation (TET) family are responsible for the oxidation of 5mC to 5-hydroxymethylcytosine (5hmC) [75]. Members of this group such as TET1, TET2, and TET3 have been shown to counterbalance the effect of 5mC by inhibiting the binding of MBP proteins. Whereas 5mC correlates positively with age and, in general, negatively with gene expression in the brain [76], 5hmC despite of correlating also positively with age [77] has been shown to associate positively with expression [61, 78]. In addition, the 5hmC mark seems to be particularly abundant in tissues with low cell renewal rates such as the cerebellum and cortex [79] where it has been shown to be highly dynamic and susceptible to age-related changes [80, 81]. The process of DNA demethylation and the enzymes catalyzing this reaction remain less well known although DNA demethylases such as the activation-induced cytidine deaminase (AID) [82] or the DNA demethylating activity of TET1 [75] have been identified. In neurons, the global balance among DNA methylation, demethylation, and hydroxymethylation determines neurobiological processes such as neural plasticity, memory, or learning, and their deregulation can be associated with neurodegenerative disorders [58].

### 2.3. Histone Modifications

In addition to DNA methylation, the conformation of the chromatin is also regulated by histone posttranslational modifications. In eukaryotic chromatin, the genomic DNA is packed around histone proteins forming the so-called nucleosome, which consists of 147 base pairs of DNA wrapped around a histone octamer containing 2 copies each of the core histones H2A, H2B, H3, and H4 [83]. The nucleosome represents the fundamental unit of eukaryotic chromatin which folds through a series of successively higher order structures to form the chromosome. Thus, the nucleosome compacts DNA and creates an added layer of regulatory control to ensure correct gene expression by determining the three-dimensional structure of DNA and its accessibility to TFs, RNA polymerases, and other DNA sequences [84]. Ultimately, the nuclear organization of the chromatin is given by the balance between condense inactive heterochromatin and open active euchromatin [85]. Ultimately, the transcriptional regulation of genes is primarily controlled by physical access of the RNA polymerase II to promoter regions. Nonetheless, gene expression is also regulated by cis-elements termed enhancers which can be distally located upstream or downstream of promoters and whose epigenetic regulation is required for gene expression [86–88]. Thus, in addition to methylation, posttranslational modifications of histones at both promoters and enhancers critically regulate the conformation of the chromatin and the transcriptional state of specific genes [89].

There are more than 100 different histone posttranslational modifications which can affect different histone amino acid residues including lysine (K), arginine (R), serine (S), threonine (T), and glutamate (E) [48]. Of these, acetylation and methylation of lysine residues are the most well-known histone modifications [90]. Enzymatically, the chemical reactions of histone acetylation/deacetylation are catalyzed by histone acetyltransferases (HAT)/deacetylases (HDAC) whereas histone methylation/demethylation are mediated by histone methyltransferases (HMT)/demethylases (HDM) which typically form chromatin-modifying complexes [91]. These histone marks are also specifically recognized by chromatin-binding proteins involved in transcriptional activation or repression and DNA replication and repair.

For instance, methylation of H3K4 can inhibit the binding of HDAC therefore favoring acetylation whereas acetylation of H3K18 facilitates the engagement of HAT [92]. Methylation and specially trimethylation of histone 3 at lysine 27 (H3K27me3)/lysine 9 (H3K9me3) have been associated with gene repression. Conversely, methylation of H3K4 normally marks active enhancers whereas acetylation of H3K4, H3K9, and H3K27 correlates with transcriptional activation [93–95]. In addition, the H3K27ac mark has been found to specifically distinguish active enhancers from poised enhancers in embryonic stem cells (ESCs) in genes which are relevant during development [86]. In general, acetylation of *ε*-amino groups of lysine residues of histones neutralizes their positive charge thereby relaxing chromatin structure [91] commonly favoring the protein binding of transcriptional activators [96]. Per contrary, histone deacetylation favors chromatin compaction and transcriptional repression [97]. Histone marks affecting H3 lysine residues have recently been associated with functional chromatin states including, in a summarized comprehensive manner, repressed regions (H3K27me3 and H3K9me3), promoter (H3K4me3), promoter/enhancer (H3K4me1 and H3K27ac), and transcriptional elongation sites (H3K36me3) [98] (Table 1). Recently, reference functional chromatin states have been defined in humans for a wide variety of tissues including the central nervous system (CNS) providing a valuable resource for future epigenetic studies [99]. In the CNS, these histone modifications have been associated with neural stem cell (NSC) maintenance, neural and glial cell type specification, homeostasis, neural plasticity, learning, memory, and aging [48].

### 2.4. Epigenetic Mechanisms during Neural Cell Differentiation

During development, the progression from pluripotent stem cells through progenitors to differentiated cells occurs through an increase of repressive histone marks, DNA methylation, and chromatin compaction [100]. These repressive epigenetic marks limit the phenotypic plasticity properties of the developing cells and therefore are essential for acquiring a differentiated cell identity [101]. Little is known about the epigenetic pattering during the development of the human brain but efforts towards its characterization are being conducted including methylome studies for at least certain cell types. Thus, a pioneer work has identified differentially methylated CpG regions associated with synaptogenesis during brain development in mouse and humans which seem to be enriched in key regulatory regions indicating their putative functional relevance [61]. In addition, this study revealed that 5hmC marks are present in fetal brain at regions that become activated by losing CG methylation and also that non-CG methylation accumulates in neurons but not in glia during this process. On the contrary, histone marks of the developing brain [102, 103] or global transcriptome alterations involved in the cell-type specification remain poorly explored [104, 105]. Yet once the neural fate program is activated, the remodeling of the chromatin is driven by cell specification signals such as TFs that interact with target sequences [106] showing binding site enrichment of the specific TFs whose activity regulates gene expression [53, 107]. Conceptually, multiple TFs acting in a coordinated manner orchestrate the remodeling of the epigenome of the differentiating neural cell to acquire specific cell phenotypes [108, 109]. These core “pioneer” TFs influence the chromatin environment by increasing the DNA accessibility to additional TFs [110] which promote cellular specification [111]. Core TFs such as OCT4, SOX2, and NANOG have been shown to be major regulators in the maintenance of pluripotency state in human embryonic stem cells (ESCs) [112, 113]. Of these, OCT4 has been shown to control the expression of H3K9me3 demethylases contributing to preserve the epigenetic marks needed for self-renewal of ESC [114]. Thus, genes transcriptionally active in ESC such as OCT4 or NANOG are characterized by H3K27ac and H3K4me3 active marks. In contrast, most key developmental genes remain inactive during ESC self-renewal and carry simultaneously bivalent chromatin marks including repressive H3K27me3 and active H3K4me1/H3K4me3 marks [115]. Among genes with bivalent marks are the HOX clusters which are master regulators of embryonic development [116] and are silenced until cell fate commitment by polycomb repressive complexes (PRC). These PRC promote chromatin condensation by adding H3K27me3 [117] while keeping a poised state of transcription. The bivalent marks become univalent active ones during ESC commitment towards neural lineage [118] by the action of specific H3K27me3 [119] and H3K4me3 demethylases [120]. In mouse NSC, bivalent marks have been shown to resolve into active H3K4me3 monovalency upon differentiation in GABAergic neurons and into repressive H3K27me3 in non-GABAergic neurons [121] indicating that genes carrying bivalent marks may lose one type of mark and become active or silenced depending on the direction of the differentiation. In general, during differentiation, a progressive closure of the chromatin occurs at loci required for differentiation [115] by a depletion of open chromatin histone marks, mainly H3 and H4 acetylation, and a simultaneous increase of repressive marks such as H3K9me3 [122, 123].

As part of the Epigenome Roadmap Project, a recent study has shown that cell specification into the three-germ layer derivatives involves dynamic changes of TFs which work coordinately in specific and sequential TF modules which are integrated by individual TFs showing similar binding preferences for common sequences [124]. Thus, specific loss of DNA methylation has been detected at target sequences due to binding of the lineage-specific TFs as well as increments of the promoter/enhancer H3K27ac mark. In the nervous system, another study characterized the TFs neural regulatory networks involved in differentiation from ESC through neuroepithelial progenitors to radial glial cells [125]. This study found that different neural stages are characterized by different epigenetic states in which distinct TFs are associated with stage-specific epigenetic changes as observed by shRNA inhibition. Thus, early stage-transition from ES to neuroepithelial progenitor showed enrichment for the promoter/enhancer H3K4me1 and H3K27ac marks whereas later transition to radial glial cell showed abundance of the promoter mark H3K4me3 [125].

### 2.5. Epigenetic Mechanisms during iPSC Reprogramming

Whereas the process of cell reprogramming means a truly epigenetic reprogramming [7], the precise epigenetic mechanisms underlying this process are only partially known. A defined set of pluripotency TFs including only four or even three reprogramming TFs, namely, OCT4, SOX2, KLF4, and MYC, have been shown to be sufficient to generate the induced pluripotent stem cell (iPSC) state [2]. These TFs are commonly known as OSKM factors (or as OSK when not including c-MYC). The expression of OSKM is needed to overcome epigenetic barriers such as the histone repressive mark H3K9m3 during cell reprogramming [126]. Once the OSKM factors are expressed and the epigenetic barriers are overcome, pluripotency is stably maintained without the need of further ectopic TF expression. Shortly after the expression of the OSKM factors, human fibroblasts initially downregulate specific markers of their somatic state to subsequently activate genes associated with pluripotency [127, 128].

To adopt the epigenome characteristic of a stem cell, the somatic cell has to erase and reorganize its chromatin epigenetic signature [129]. This process involves the genomewide resetting of histone marks which occurs immediately after the induction of OSKM factors [2, 130–132]. Subsequently, the DNA demethylation of promoter regions of pluripotency genes such as NANOG, SOX2, and OCT4 is mediated by activation-induced cytidine deaminases (AID) which are required at later stages of the reprogramming process [82, 130, 133]. Yet DNA demethylation can also occur early since AID is needed to demethylate the OCT4 promoter in fibroblasts and to initiate the process of nuclear reprogramming towards pluripotency [82]. Recent studies have suggested that the OSK TFs act as pioneer factors in loosening the chromatin into more open accessible forms and allowing the activation of genes relevant to the establishment and maintenance of the induced pluripotent state [134]. The initial histone posttranslational changes induced by OSKM include acetylation, methylation, phosphorylation, and ubiquitination of histones. These histone posttranslational changes are catalyzed by HAT and HMT (also known as the “writers”) and HDAC and HDM (known as the “erasers”) [52] which act, respectively, as coactivators or corepressors of the OSKM factors [126]. Among the earliest processes, an increase of the H3K4me2 mark occurs at promoter and enhancer regions of the genes involved in pluripotency which are enriched for binding sites of the OSKM factors and lack the H3K4me1 and H3K4me3 active marks [130]. To achieve pluripotency induced by OSKM, recent studies have shown that there are three groups of epigenetic targets. First, somatic genes with open chromatin states showing DNase I hypersensitivity and active histone marks H3K4me2 and H3K4me3 are readily accessible to OSKM to be downregulated [134]. Second, distal regulatory elements showing DNase I hypersensitivity and the enhancer mark H3K4me1 act as permissive enhancers that, after the binding of OSKM, are associated with promoters eliciting nucleosome depletion, chromatin relaxation, and transcriptional activation of lineage-specific genes [135]. A third group of OSKM targets encompasses core pluripotency genes containing heterochromatic regions enriched for the repressive mark H3K9me3 in which the binding of OSKM leads to the repression of non-lineage-specific genes [136].

The epigenetic remodeling of chromatin during reprogramming towards pluripotency also requires changes in DNA methylation. Although DNA methylation is considered as the most stable epigenetic modification conferring permanent gene silencing during development [126], histone modifications have been shown to typically antedate changes in DNA methylation during development [109] and consistently this hierarchy of events has also been observed in reprogramming [133]. Demethylation of pluripotency genes is crucial for faithful reprogramming, and although demethylation can occur either by passive or active mechanisms [137], active demethylation catalyzed by specific enzymes has been shown to play a more important role in the induction of pluripotency [126]. In addition, a progressive decrease of DNA methylation and of the H3K27me3 repressive mark at promoters of genes relevant to conversion occurs throughout reprogramming [133]. Although these changes take place almost exclusively at CpG islands of initiating loci at the beginning of reprogramming process, they later expand outside CpG islands to affect other regions [138]. During reprogramming, inefficient DNA demethylation or remethylation has been associated with “epigenetic memory”; that is, the partial retention in iPSC of epigenetic and transcriptional patterns of the somatic cell type of origin which as consequence may limit the differentiation properties to generate specific cell-type derivatives favoring the generation of certain cell types over others [139]. This epigenetic memory has been linked to the failure to reverse repressive epigenetic marks associated with cell fate commitment [101]. To date, epigenetic memory has been regarded as intrinsic limitation of iPSC permitting pluripotency but not totipotency.

## 3. Epigenetic Research of Neurodegenerative Disorders Using iPSCs

From a technical point of view, ESC represents an ideal tool to investigate development and model human disease as they provide a virtually endless resource of cells of interest given their high self-renewal and differentiation capacity. However, the use of ESC has been limited by ethical issues since current isolation protocols of ESC from the blastocyst inner cell mass imply the destruction of the embryo. In this scenario, in vitro generation of iPSC has contributed to overcome at least in part such an obstacle. Here, we will review the potential of iPSC models as promising cell systems to perform epigenetic research of neurodegenerative disorders in the context of human postmortem brain tissues and animal models which can also implement this new venue of research.

### 3.1. Genomewide Methylation Studies in Patient Postmortem Brain Tissues

A recent study investigated the methylome of AD in cortex tissue grey matter using a large number of prospectively collected autopsied brains from patients and controls [140]. This study identified differential DNA methylation in 11 CpGs which correlated with AD pathology as assessed by the burden of neuritic amyloid plaques and with RNA expression. Six of the identified differentially methylated genes connected to a known genetic network of AD susceptibility. Among these, methylation differences in the* ANK1* gene were further confirmed in an independent analysis of entorhinal cortex, which is a primary site of AD pathology, as well as in other affected areas including the superior temporal gyrus and the prefrontal cortex [141]. In PD, one genomewide association study (GWAS) identified new genetic variants associated with disease and, for a subset of genes, it also found differential methylation levels in PD frontal cortex and cerebellum which overlapped with previously reported genetic associations [142]. Another genomewide DNA methylation study in PD frontal cortex also identified distinct methylation patterns in PD affecting genetic polymorphisms associated with PD and, interestingly, these differential methylation patterns correlated in brain and blood samples [143]. Altogether, these studies in AD and PD provide the proof-of-concept that epigenetic deregulation occurs in neurodegenerative disorders and encourage the use of iPSC-based models to conduct epigenetic research in these diseases. However, DNA methylation changes from these studies were detected despite of the heterogeneous mix of brain cell types, and therefore it is possible that overall epigenetic differences may be underestimated. Similarly, it would be expectable that epigenetic changes associated with disease could be potentially identified using iPSC-based models albeit of the cell population heterogeneity which is inherent to these models. Yet in this scenario, iPSCs models offer the opportunity to characterize the epigenetic profiles of specific cell populations by using techniques such as fluorescence-activated cell sorting (FACS) as recently shown for the transcriptome characterization of mouse iPSC-derived DAn [144].

### 3.2. Lessons from Epigenetic Studies in Mouse Models

A recent RNA-seq study in the Ck-p25 mouse model of AD identified gene expression upregulation of immune system genes and downregulation of genes associated with neuronal function [145]. Similar findings were also reported in human AD postmortem hippocampus [146]. These expression changes correlated with the epigenomic status of promoters, enhancers, and polycomb-repressed regions which showed a specific depletion of neuronal promoter and enhancer marks. Interestingly, this study demonstrated a strong conservation of gene expression and epigenomic signatures between human and mouse with a specific enrichment of AD-associated loci in enhancer orthologs. Similarly, in PD the epigenomic [147] and transcriptomic [144] signatures of a mouse model of iPSC-derived DAn have been deeply characterized but similar studies in PD human iPSC-based models are still missing. Another study in PD compared the transcriptome and the methylome of primary embryonic mesodiencephalic DAn from the Pitx3^Gfp/+^ knock-in mouse as well as iPSC-derived DAn generated upon embryonic fibroblast reprogramming [148]. PITX3 is a highly specific maker of DAn of the substantia nigra and FACS analysis based on the Pitx3-GFP reporter revealed that although mouse iPSC-derived DAn largely adopted highly similar global gene expression and DNA methylation patterns as their in vivo counterparts, they also showed deviations including intermediately methylated neural loci (40–60% methylation) whose role yet remains to be elucidated. Altogether, these studies in AD and PD illustrate a scenario in which epigenetic research relevant to neurodegenerative diseases is more advanced in iPSC-based models from mouse than humans due to availability reasons. Yet achievements of these mouse epigenetic studies can be useful for epigenetic research using patient-derived iPSC-based models since mouse studies can provide valid technical data which may help to prevent pitfalls in designing experiments using human iPSCs as well as to generate novel epigenetic knowledge to be explored in human models genuine to the patients.

### 3.3. iPSC Models for Epigenetic Research of Neurodegenerative Disorders

**Figure 1:**
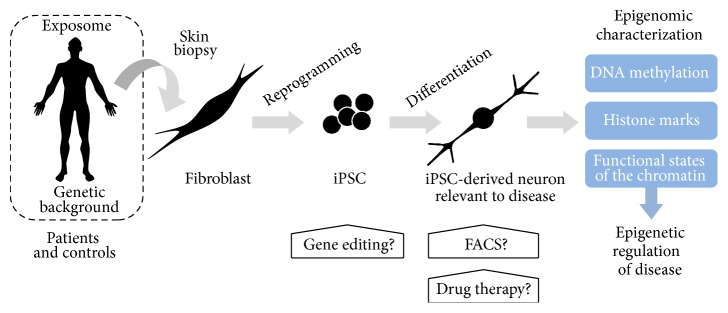
Possible experimental design of epigenomic characterization of neurodegenerative diseases using patient-specific iPSC-derived neural models.

Recently, well-established protocols have been elaborated to generate patient-derived disease-relevant cell types upon iPSC reprogramming (comprehensively reviewed in [149]). The specific cell types obtained by these methods include iPSC-derived DAn in PD [21–30], MN in ALS [31–37], striatal medium spiny neurons in HD [31–37], or neurons in AD [42–45]. Although these protocols have been steadily improved by increasing reprogramming and differentiation efficiencies, cell heterogeneity accompanying disease-relevant cell types is still inherent to current iPSC models. This accompanying cell heterogeneity can act as a potential confounder in epigenetic research but, yet, if affecting in an equal manner to iPSC from patients and controls, it may also lead to an underestimation of the observed epigenetic differences, as recently suggested in postmortem epigenetic studies analyzing heterogeneous mix of brain cells [140, 141]. Still this cell heterogeneity should be appropriately controlled for epigenetic studies by performing FACS isolation to deliver pure cell populations prior to the epigenetic analyses [144, 148]. Alternatively, it could also be possible to control the variability caused by cell heterogeneity by studying the epigenetic profile of iPSC-derived neural types nonenriched in the specific neural type of interest, for example, iPSC-derived neural cultures nonenriched in DAn as a control population for a DAn study in PD. Thus, if differences appear only in DAn but not in other cultures nonenriched in DAn, the identified epigenetic differences would be attributable to DAn (Fernández-Santiago et al., unpublished data). Yet despite of current technical challenges, cellular reprogramming provides conceptually a unique opportunity to generate in vitro human models that will permit to investigate epigenetic regulation and alterations of functional states of the chromatin related to neurodegenerative diseases [52]. Recently, the epigenetic signature from 111 human tissues has become publically available including multiple brain regions such as the hippocampus or the substantia nigra which are relevant to AD and PD, respectively [99]. This large multicentre study has implemented the reference human genome sequence and is expected to set the basis for future studies on epigenetic variation and its role in human disease by providing reference maps of histone modifications and DNA methylation, as well as global RNA expression data. This information will prove instrumental to investigate specific epigenetic alterations and to model in vitro novel epigenetic disease mechanisms using currently available patient-derived iPSC-based models of neurodegenerative diseases which up to date have not been epigenetically characterized [150]. Interestingly, iPSC-derived neural models preserve the genetic background of the patient and this is relevant since the disease-associated genetic variants were previously shown to be enriched in tissue-specific epigenomic marks suggesting an overlap of genetic and epigenetic alterations which may be associated with human disease [99]. In addition, iPSC-derived neural models can virtually offer the opportunity to recapitulate the exposome or environmental history of the individual that may be relevant complex diseases with an expected large environmental contribution such as AD, PD, or ALS and also to their monogenic forms (Figure 1).

### 3.4. iPSCs Models for Epigenetic Research in Monogenic versus Sporadic Forms of Neurodegenerative Disorders

With the exception of HD which is a largely monogenic disease, most of the patients with other neurodegenerative disorders such as AD, PD, or ALS are considered sporadic or idiopathic. In these cases, the disease is expectably driven by the cumulative and/or synergistic effect of genetic risk variants together with largely unknown environmental conditions [151–153] whose effect could eventually be reflected in the epigenome of the patients. To date, although iPSC-based disease modeling has been preferentially performed in mutation-caused monogenic forms of neurodegenerative disease, recent studies in AD and PD have set the proof-of-concept that iPSC-derived models from sporadic patients can exhibit molecular alterations similar to those changes detected in monogenic patients [28, 42, 43]. In monogenic cases, iPSC-based systems offer the attractive possibility to perform gene editing [29] contributing to the elucidation of the molecular events triggering disease through the analysis of the effect of specific pathogenic mutations. Unfortunately, this approach is not to be feasible for sporadic forms due to the polygenic effect of the multiple genetic risk factors which are expected to be involved in the sporadic disease. Despite this inconvenience, iPSC-derived systems have proved efficient to model sporadic disease as, for example, in PD [154] but, yet, it has not clarified the underlying mechanisms by which these iPSC-derived models from sporadic patients can develop disease phenotypes. It can be hypothesized that since iPSC-based neural models preserve the genetic background from the patient, derived neurons also carry the specific set of susceptibility genetic variants which could ultimately trigger the disease initiating pathogenic changes. Alternatively, biological alterations and damages could already be present in the primary fibroblast as consequence of the interaction of the genetic background and environmental factors but their full pathogenic effect might only be observed in the appropriate context of the disease-relevant neural cell types. Supporting this view, biological alterations have been recently described in fibroblast from sporadic cases with PD or AD [155, 156], thus reinforcing the idea of latent molecular defects which can be present in the somatic cells. Yet the genetic or epigenetic nature of these potentially latent molecular defects in the somatic cells from sporadic cases has so far not been explored into detail in neurodegenerative diseases. In addition, interactions of genetic and epigenetic factors represent an important field of investigation in complex disorders [157]. In this scenario, patient-specific iPSC-derived neural cells could represent useful models able to not only capture the subject genetic background but also potentially recapitulate the environmental exposome of the individual through the epigenome (Figure 1). Accordingly, iPSC-based neural models are expected to be helpful for investigating epigenetic changes of the sporadic forms of neurodegenerative diseases where the environment is supposed to play a more prominent role. However, the complexity of these multifactorial diseases is expected to be high, especially when taking into account the presence of possible interactions between genetic risk variants and their methylation status that could ultimately modify their pathogenicity [157]. Under this view, iPSC-derived neural models open new research venues to investigate epigenomic changes associated with neurodegenerative diseases and most especially with their sporadic forms.

### 3.5. Environmental Epigenomics in Complex Neurodegenerative Diseases

Environmental conditions include the exposition of an individual to drugs, toxins, metabolites, or other external stimuli. However, the environment can also be considered as the single cell microenvironment encompassing external cellular stimuli, inflammatory responses, or signaling from nearby cells. Yet both of these macro- and microenvironmental conditions have been shown to contribute to the modification of the epigenome by ultimately inducing interoceptive cell signaling cascades [158]. These environmental conditions have also been shown to contribute to the epigenetic drift observed in monozygotic twins who accumulate diverging epigenomic changes over time [56]. Among metabolites modulating the epigenome, folic acid has been shown to remodel the chromatin conformation at neural promoters during neural tube development indicating that environmental exposition to chemical cues can be associated with epigenetic regulation relevant to the nervous system [159]. In addition, cumulative evidence has shown that other compounds interfering with epigenetic control during early development are suspected to consequently cause other neural defects later in life [160]. Thus, epigenetic research could provide novel mechanistic paradigms for developmental toxicology studies in late-onset diseases like AD or PD [161, 162] where epigenetic changes could mediate the transition from an early insult caused by chemical compounds to an adverse effect on the developing nervous system [160]. Several works have also revealed associations between early-life exposure to pesticides and PD [163] but the epigenetic involvement in this pathogenic process is yet unclear. As examples of environmental factors potentially triggering neurodegenerative diseases such as PD later in life, cell culture studies have shown that exposure to several neurotoxicants such as methyl mercury (MeHg) impairs the formation of DAn or reduces their neuritic growth [164, 165]. In adults, pesticide-induced hyperacetylation of histones leading to chromatin decondensation and nonspecific transcriptional upregulation has been linked to PD [166]. For example, Paraquat another pesticide acting as neurotoxin in PD has been associated with hyperacetylation of histones [167]. In addition, time-dependent increase of H3 and H4 hyperacetylation induced by environmental toxins such as the insecticide dieldrin has also been associated with the pathophysiology of PD [168]. Moreover, recent studies have demonstrated the mediation of specific epigenetic mechanisms in promoting axonal regeneration after spinal cord injury providing further evidence of the influence of environmental cues in the epigenome of the individual [169]. In this context, patient-derived iPSC-based cellular models of neurodegenerative disease could represent a valid tool to explore the effect on the subject epigenome of candidate environmental factors identified in epidemiologic studies. Results from these studies may ultimately contribute to deciphering the pathophysiological processes associated with environmental conditions in neurodegenerative disorders by the identification of specific underlying epigenetic mechanisms.

## 4. Epigenetic Therapeutic Targets in Neurodegenerative Disorders

In principle, iPSC models are ideally suited for drug development due to their limitless self-renewal capacity allowing the production of large quantities of cells and to their high differentiation properties into disease-specific cell types. However, iPSC models have not yet been extensively used in large-scale drug screenings in neurodegenerative disorders due to the clonal variation associated with stochastic gene mutation [170] and also due to the difficulties in controlling for correct efficiency differentiation when using large amounts of clones. Thus, although studies of several thousands of compounds have been published in ALS [171, 172] or AD [173], large-scale drug studies using iPSC are still an ongoing area of development. On the contrary, strategies using a limited number of candidate therapeutic drugs have been successfully tested in other diseases [174]. This approach seems to be more feasible at present for neurodegenerative diseases as recently shown in iPSC models of AD [175] and ALS [35].

Epigenetic drugs currently explored in human disease models include most prominently histone deacetylation inhibitors, DNA methylation inhibitors, and histone acetylation activators [158]. Conceptually, HAT and HDAC maintain the balance of correct acetylation marking of histone lysine residues upon acetyl-coenzyme A as donor of acetyl groups. HAT act by enhancing the DNA accessibility for TF binding and increasing gene expression while on the contrary HDAC have the opposite effect by attenuating transcription. These acetylation balances determine cell survival and homeostasis whereas imbalances are related to pathological conditions [176, 177]. In neurodegenerative disorders, recent reports have suggested that the deregulation of histone acetylation levels could be modulated by epigenetic drugs [178, 179]. Thus, drugs activating HAT [180] as well as HDAC inhibitors have been shown to improve neuroprotection and synaptogenesis [181, 182]. Moreover, epigenetic drugs modulating HAT or HDAC activity have been shown to alleviate pathological symptoms in experimental models of PD, AD, and HD by reverting abnormal gene repression associated with disease [183–186]. Yet different drugs may be needed for different aspects of disease [158] since, for example, in ALS a HAT inhibitor called anacardic acid has been proved effective in downregulating abnormal gene expression and rescuing ALS MN phenotype [35]. Valproic acid (VA) is one HDAC inhibitor enhancing H3 acetylation which has been shown to be neuroprotective against MPTP-induced neurotoxicity in PD mouse models [187]. The neuroprotective effect of VA has been demonstrated to be mediated by glial cell-derived factor (GDNF) and brain-derived neurotrophic factor (BDNF) signaling in DAn models from rats [188]. ESCs retinoic acid (RA), which is a determinant for anteroposterior patterning of the developing CNS, has been shown to have a similar effect as HDAC inhibitors by increasing histone acetylation levels and upregulating gene expression of its targets [189].

Specifically in PD, other studies illustrate that epigenetic drugs can be useful to modulate disease aspects related to epigenetic deregulation. Thus, a recent study in a human DAn model and mouse organotypic brain slice cultures has shown that the treatment with the HDAC inhibitor sodium butyrate (NaBu) upregulates the expression of oxidative stress-sensitive protein kinases (PKCs) and augments DAn apoptotic cell death [184]. Since the effect of this HDAC inhibitor directly leads to H4 hyperacetylation, this study supports the role of HDAC deregulation in PD and identifies novel potential epigenetic therapeutic targets. In addition, DNMT inhibitors such as 5-aza-2′-deoxycytidine (5-aza-dC) have been shown to induce the expression of tyrosine hydroxylase (TH), the synthesis of dopamine, and also the expression of alpha-synuclein [190]. Moreover, levodopa-induced dyskinesia which is a major side effect of the levodopa treatment in PD patients has been associated with histone deacetylation in PD animal models [191] suggesting a possible role of histone acetylating drugs for the treatment of dyskinesias [192]. In general, if levodopa is proved to act through an epigenetic pathway, traditional treatments should be revisited to elucidate the novel epigenetic aspects and to design novel and more specific medications [158].

In summary, let alone that epigenetic research of neurodegenerative disorders is an emerging field, the identification of epigenetic therapeutic targets for AD, PD, or ALS is at its infancy. In this scenario, iPSC-based models may be useful not only to detect epigenetic changes associated with these diseases but also to explore the ability of candidate epigenetic drugs to correct epigenetic alterations and to design novel therapeutic strategies. To date, this goal seems to be technically feasible only for small or medium scale drug studies or for very specific drugs. Yet future patient-specific iPSC-based systems using improved cell reprogramming protocols are expected to pave the way out for epigenetic research ultimately intending the delivery of personalized epigenetic therapies.

## 5. Future Perspectives and Open Questions

Although recent works have provided evidence about the involvement of epigenetic changes in neurodegenerative diseases and supported the use of iPSC-derived neural models to explore epigenetic alterations in these disorders, several questions remain to be answered.

A first question is whether iPSC-derived neurons really mimic the epigenetic and expression features occurring in the affected brain areas of the patients. Epigenetic changes relevant to neurodegenerative diseases are expected to reflect the complex interaction of genetic background, environmental factors, and gene expression in the context of the brain. Accordingly, it would be necessary to assess whether iPSC-derived neurons obtained from patient fibroblasts do faithfully recapitulate the molecular events occurring in the complex cell microenvironment of the brain thus representing good models of disease. A direct approximation would encompass the comparison and determination of the level of coincidence of epigenetic marks in iPSC-derived neurons and in their patient brain cell counterparts as investigated in a recent study [148]. However, defining the epigenetic alterations related with disease remains challenging since usually postmortem brain tissues are only available after many years of disease evolution and also after important cell loss of the neurons targeted by disease. In addition, it is also essential to determine whether changes in the epigenomic profile, the gene expression patters, the protein composition, and the overall neuron performance could represent on themselves initial changes triggering disease or alternatively secondary physiological changes of the neurodegenerative process [193]. Fairly, this is an important application of iPSC-derived cells models of neurodegenerative diseases which could help to identify early alterations occurring in nervous tissues [194]. These cells can show neural networks and are functional in terms of biochemistry, electrophysiology, and synaptic transmission as previously described [28, 42]. Yet it remains to be demonstrated whether the epigenetic alterations detected in iPSC-derived neurons could represent pathological epigenetic changes associated with disease or alternatively whether they could be beneficial compensatory changes in response to disease injury caused by other molecular mechanisms.

Second, assuming that iPSC systems represent good disease models for neurodegenerative diseases, a new venue would be open to explore epigenetic changes in specific genetic loci by using high resolution whole-genome methodologies, including commercially available methylation arrays, whole-genome bisulphite sequencing, or whole-genome histone marks analyses in patients and controls. In order to implement our comprehension of disease mechanisms, these data could be analyzed by integrative biology methods interrogating the epigenome, the transcriptome, and the known risk genetic loci detected in genomewide association studies (GWAS) [195–197]. In this way, GWAS have identified hundreds of genetic risk variants for neurodegenerative diseases like AD or PD [198–200] which are located in different genes loci affecting aetiological pathways involved in disease but with subtle effects on disease susceptibility. However, it is important to note that more of the risk polymorphisms identified until present by GWAS has not immediately provided functional insights and also that most of the risk variants cannot always be clearly assigned to target genes since many variants are located in noncoding or intergenic regions [201]. Thus, to improve our understanding of disease risk mechanisms, it is possible to correlate cis- or trans-located genetic risk polymorphisms, gene activity as determined by transcripts quantification [202, 203], and CpGs methylation differences at specific loci [204]. These genetic variants can affect gene expression activity by altering the affinity of DNA binding TFs [205] leading to differential methylation patterns and if located in enhancers regions they can also alter the expression of distal target genes [204]. Thus, this combined approach associating specific candidate genetic polymorphisms, gene expression changes, and differential methylated CpGs could help to gain insight into the functional consequences of genetic variants associated with disease risk and to facilitate the interpretation of data from GWAS studies [206], for example, by restricting the analysis of risk candidate polymorphism in GWAS to those associated with differences in methylation levels in disease-targeted cells [197]. Thus, we anticipate that epigenetic and expression alterations detected in iPSC-derived models of neurodegenerative diseases will serve as a functional system to reinterpret the genetic risk loci associated with these diseases by implementing the knowledge of the pathogenic mechanisms associated with the risk genetic loci.

Third, assuming that these neurons recapitulate causative epigenetic alterations, a new opportunity will be offered to explore the capacity of different epigenetic-modifying drugs to modulate pathologic epigenetic patterns and then observe their respective functional effects and establish cause-effect mechanisms. In this way, iPSC-derived neurons could represent a valid tool to test potential therapeutic drugs reversing the pathological epigenetic changes and to monitor disease-relevant processes such as alpha-synuclein or amyloid deposition, autophagy alterations, or mitochondrial dysfunction. In summary, iPSC-derived neurons models have promising perspectives and open new avenues for biological mechanistic studies, drug discovery and testing, and clinical therapy of neurological disorders related with epigenetic changes.

## Figures and Tables

**Table 1:** Comprehensive summary of histone epigenetic marks and corresponding functional states of the chromatin.

Histone epigenetic mark	Chromatin state
H3K27me3	Repressed
H3K9me3	Repressed
H3K4me3	Promoter
H3K4me1	Promoter and enhancer
H3K27ac	Promoter and enhancer
H3K36me3	Transcriptional elongation
CTCF-binding sites	Insulator
